# Curcumin-Loaded Apotransferrin Nanoparticles Provide Efficient Cellular Uptake and Effectively Inhibit HIV-1 Replication *In Vitro*

**DOI:** 10.1371/journal.pone.0023388

**Published:** 2011-08-22

**Authors:** Upendhar Gandapu, R. K. Chaitanya, Golla Kishore, Raju C. Reddy, Anand K. Kondapi

**Affiliations:** 1 Department of Biotechnology, University of Hyderabad, Hyderabad, India; 2 Department of Biochemistry, School of Life Sciences, University of Hyderabad, Hyderabad, India; 3 Centre for Nanotechnology, University of Hyderabad, Hyderabad, India; 4 Department of Medicine, Division of Pulmonary, Allergy and Critical Care Medicine, Emory University, Atlanta, Georgia, United States of America; University of Helsinki, Finland

## Abstract

**Background:**

Curcumin (diferuloylmethane) shows significant activity across a wide spectrum of conditions, but its usefulness is rather limited because of its low bioavailability. Use of nanoparticle formulations to enhance curcumin bioavailability is an emerging area of research.

**Methodology/Principal Findings:**

In the present study, curcumin-loaded apotransferrin nanoparticles (nano-curcumin) prepared by sol-oil chemistry and were characterized by electron and atomic force microscopy. Confocal studies and fluorimetric analysis revealed that these particles enter T cells through transferrin-mediated endocytosis. Nano-curcumin releases significant quantities of drug gradually over a fairly long period, ∼50% of curcumin still remaining at 6 h of time. In contrast, intracellular soluble curcumin (sol-curcumin) reaches a maximum at 2 h followed by its complete elimination by 4 h. While sol-curcumin (GI_50_ = 15.6 µM) is twice more toxic than nano-curcumin (GI_50_ = 32.5 µM), nano-curcumin (IC_50_<1.75 µM) shows a higher anti-HIV activity compared to sol-curcumin (IC_50_ = 5.1 µM). Studies *in vitro* showed that nano-curcumin prominently inhibited the HIV-1 induced expression of Topo II α, IL-1β and COX-2, an effect not seen with sol-curcumin. Nano-curcumin did not affect the expression of Topoisomerase II β and TNF α. This point out that nano-curcumin affects the HIV-1 induced inflammatory responses through pathways downstream or independent of TNF α. Furthermore, nano-curcumin completely blocks the synthesis of viral cDNA in the gag region suggesting that the nano-curcumin mediated inhibition of HIV-1 replication is targeted to viral cDNA synthesis.

**Conclusion:**

Curcumin-loaded apotransferrin nanoparticles are highly efficacious inhibitors of HIV-1 replication *in vitro* and promise a high potential for clinical usefulness.

## Introduction

Curcumin, (diferuloyl methane) is a polyphenol obtained from the rhizome of the herb *Curcuma longa* (turmeric). Curcumin has been shown to exhibit anti-oxidant [1], anti-inflammatory [2], anti-microbial [3] and anti-carcinogenic [4] activities. It also is hepato- and nephro-protective [5], [6], suppresses thrombosis [7], protects against damage due to myocardial infarction [8] and exhibits hypo-lipidemic [9] and anti-rheumatic activities [10]. Various animal models and human studies have established that curcumin is extremely safe even at very high doses (12 g/day). In spite of its efficacy and safety, curcumin has not yet been utilized as a therapeutic agent due to its limited bioavailability, a result of poor absorption, high rate of metabolism and rapid systemic elimination [11]. Almost the entire dose of orally administered curcumin is excreted in the faeces. At high doses, the plasma contains nanomolar concentrations of the parent compound and glucuronide together with sulfate conjugates [12], [13]. Enhanced bioavailability should bring this natural product to the forefront of promising therapeutic agents.

Numerous approaches were tried earlier that aimed at improving the bioavailability of curcumin. These include usage of adjuvants which can block metabolic pathways of curcumin [14] and encapsulation in liposomes or nanoparticles of various compositions [15], [16]. Though these delivery systems are biocompatible, they mostly lack target specificity. In order to enhance specificity, many drug-loaded materials are conjugated with apotransferrin/transferrin proteins [17], [18], which are abundantly expressed in actively proliferating cells. Encapsulation with these proteins enables preferential localization into target cells through receptor-mediated endocytosis [19]. This apotransferrin nanoparticle-drug delivery system also provides all the general advantages offered by nano-formulations such as appropriate size for cellular uptake, excellent water dispensability and improved intracellular localization. HIV-1 infected cells are known to express transferrin receptors, which bind transferrin or apotransferrin and transport it into the cell [20]. These receptors could be targeted for ligand-mediated transport of curcumin into the infected cells. In the present study, we formulated curcumin-loaded apotransferrin nanoparticles (nano-curcumin) using a sol-oil technique. These curcumin loaded nanospheres were then assessed for their efficiency of cellular uptake and cytotoxicity in T-cells. The nano-curcumin formulation was further evaluated for its efficacy to inhibit HIV-1 replication. The results clearly highlight the advantage of this delivery system over direct soluble-curcumin administration.

## Results

### Preparation of curcumin-loaded apotransferrin nanoparticles

**Figure 1:**
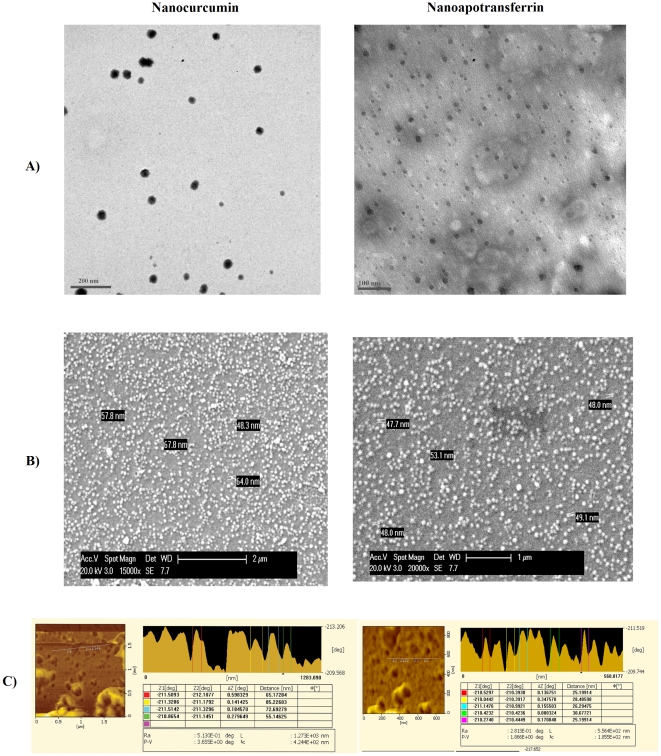
Curcumin loading increases size of apotransferrin nanoparticles. The preparations of curcumin-loaded apotransferrin nanoparticles (nano-curcumin; left) and apotransferrin nanoparticles without curcumin (nano-apotransferrin; right) were examined by A) TEM B) SEM and C) AFM as indicated.

Curcumin-containing apotransferrin nanoparticles were prepared using sol-oil chemistry as described in materials and methods section. Transmission electron microscopy (TEM) analysis showed that the particles were nearly uniform in size and spherical in shape. This technique also confirmed the increase in diameter of loaded particles (Fig. 1A). The size of pure apotransferrin nanoparticles as assessed by scanning electron microscopy (SEM) ranged from 45–55 nm, increasing to 55–70 nm after curcumin loading (Fig. 1B). The surface morphological analysis of particles using atomic force microscopy (AFM) showed significant projections, which might contribute to the molecular recognition of particle by the receptor (Fig. 1C). The proteinaceous nature of nanoparticle surface was confirmed by their sensitivity to pH 5–6. Drug loading was 50% with 500 µg of curcumin/mg of protein upon complete saturation.

### Cellular uptake of curcumin following nano-curcumin administration

**Figure 2:**
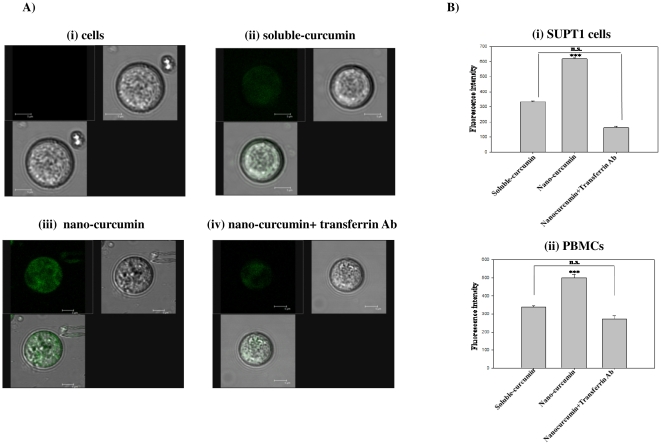
Nanoparticle formulation increases curcumin uptake, which is inhibited by transferrin receptor blockade. A) SUP-T1 cells were incubated for 1 h with curcumin formulations as indicated, then examined by confocal microscopy. (i) Cells without curcumin; (ii) 1 µM sol-curcumin; (iii) 1 µM nano-curcumin; or (iv) 1 µM nano-curcumin in the presence of transferrin receptor antibody (100 ng/ml). Each panel contains three images: fluorescence, bright field and merged. B) SUP-T1 cells (i) or stimulated PBMCs (ii) were incubated for 1 h with curcumin formulations, after which intrinsic fluorescence of intracellular curcumin was determined quantitatively by fluorometric analysis. Cells were treated with 5 µM sol-curcumin, 5 µM nano-curcumin, or 5 µM nano-curcumin in the presence of antibodies to the transferrin receptor (TrR-Ab; 100 ng/ml). All the values are normalized to that obtained from SUPT1 cells. Error bars indicate standard deviation (SD). ***, *P*≤0.001 compared to sol-curcumin; n.s.: non-significant.

Cellular uptake of curcumin upon incubation with nano-curcumin was monitored by confocal microscopic analysis of the compound's intrinsic green fluorescence. Intracellular localization of curcumin was enhanced in nano-curcumin treated cells compared to those treated with soluble-curcumin (Fig. 2A ii and iii), indicating that apotransferrin encapsulation significantly increases cellular uptake of curcumin. The curcumin localization in overall population of SUPT1 cells was given in Fig. S1. To determine whether the enhanced uptake of apotransferrin-encapsulated curcumin requires interaction with the transferrin receptor, we incubated the cells concurrently with nano-curcumin and antibody to the human transferrin receptor. The observed decrease in intracellular curcumin fluorescence (Fig. 2A iv) suggests that nano-curcumin uptake results from endocytosis mediated by the transferrin receptor. Similar results were seen when intracellular curcumin accumulation was quantified fluorimetrically in experiments conducted with SUPT1 and stimulated PBMCs (Fig. 2B). These results confirm that cellular uptake of curcumin is significantly enhanced by apotransferrin encapsulation and that this improved uptake is mediated by the transferrin receptor in both SUPT1 (Fig. 2B i) and PBMCs (Fig. 2B ii).

### Cellular retention of curcumin following nano-curcumin administration

**Figure 3:**
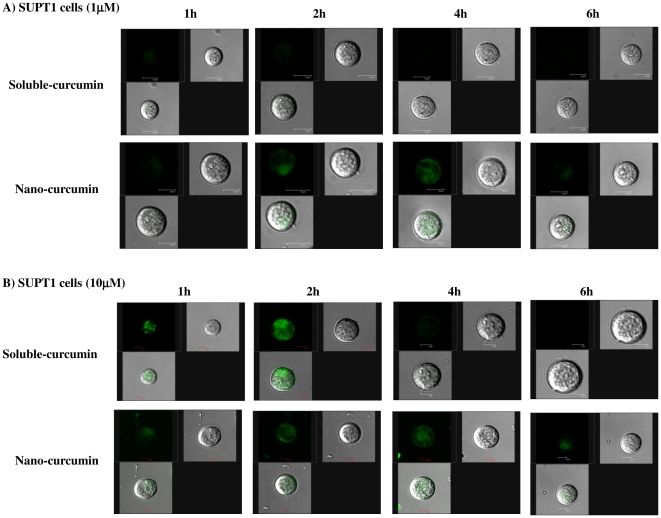
Nanoparticle formulation exhibits increased cellular retention in SUP-T1 cells. Cells were incubated with 1 µM (Panel A) and 10 µM (Panel B) sol-curcumin and nano-curcumin and examined by confocal microscopy at time points of 1, 2, 4 and 6 h. Each panel contains three images: fluorescence, bright field and merged.

**Figure 4:**
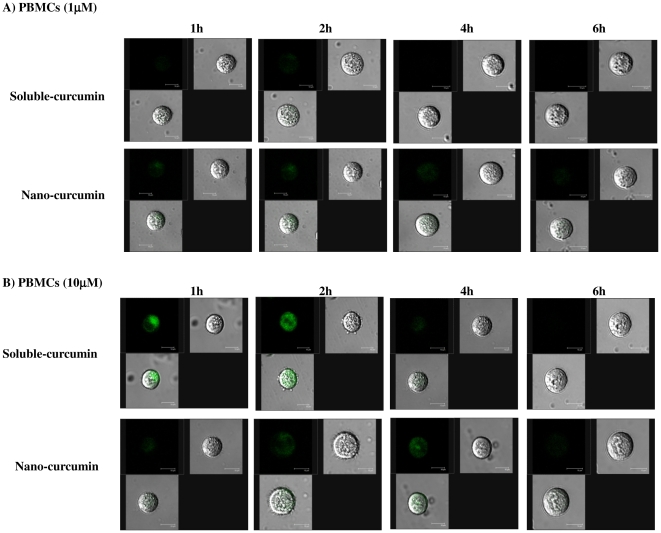
Nanoparticle formulation exhibits increased cellular retention in stimulated PBMCs. Cells were incubated with 1 µM (Panel A) and 10 µM (Panel B) sol-curcumin and nano-curcumin and examined by confocal microscopy at time points of 1, 2, 4 and 6 h. Each panel contains three images: fluorescence, bright field and merged.

Cellular uptake and elimination of curcumin upon treatment with sol- or nano-curcumin (1 and 10 µM) was assessed by confocal microscopic analysis at different time points (1, 2, 4 and 6 h respectively). Soluble-curcumin was taken up quickly by the cells, peaking at 2 h, but was rapidly eliminated out and was essentially gone by 4 h (Figs. 3 and 4). Nano-curcumin, by contrast was both taken up and released more slowly with a peak at 4 h and ∼50% of the drug still present at 6 h (Figs. 3 and 4). Further, the results confirmed that nano-cucumin exhibits a time-dependent intracellular localization of curcumin (in both SUPT1 cells PBMCs) that is stable for almost the full 6 h. In contrast, soluble-curcumin is taken up rapidly, especially at the higher concentration but disappeared from the cells quickly. This gradual and stable uptake of nano-curcumin is characteristic of receptor-mediated transport. Moreover, it is important to note that uptake at 2 h is similar for the 1 and 10 µM concentrations of nano-curcumin (Figs. 3 & 4 A and B) while uptake is significantly higher for 10 µM than for 1 µM nano-curcumin at 6 h (Figs. 3 & 4 A and B).

These results indicate that cells retain curcumin for longer periods following treatment with nano-curcumin than with sol-curcumin.

### Cytotoxicity of nano-curcumin mediated delivery

**Figure 5:**
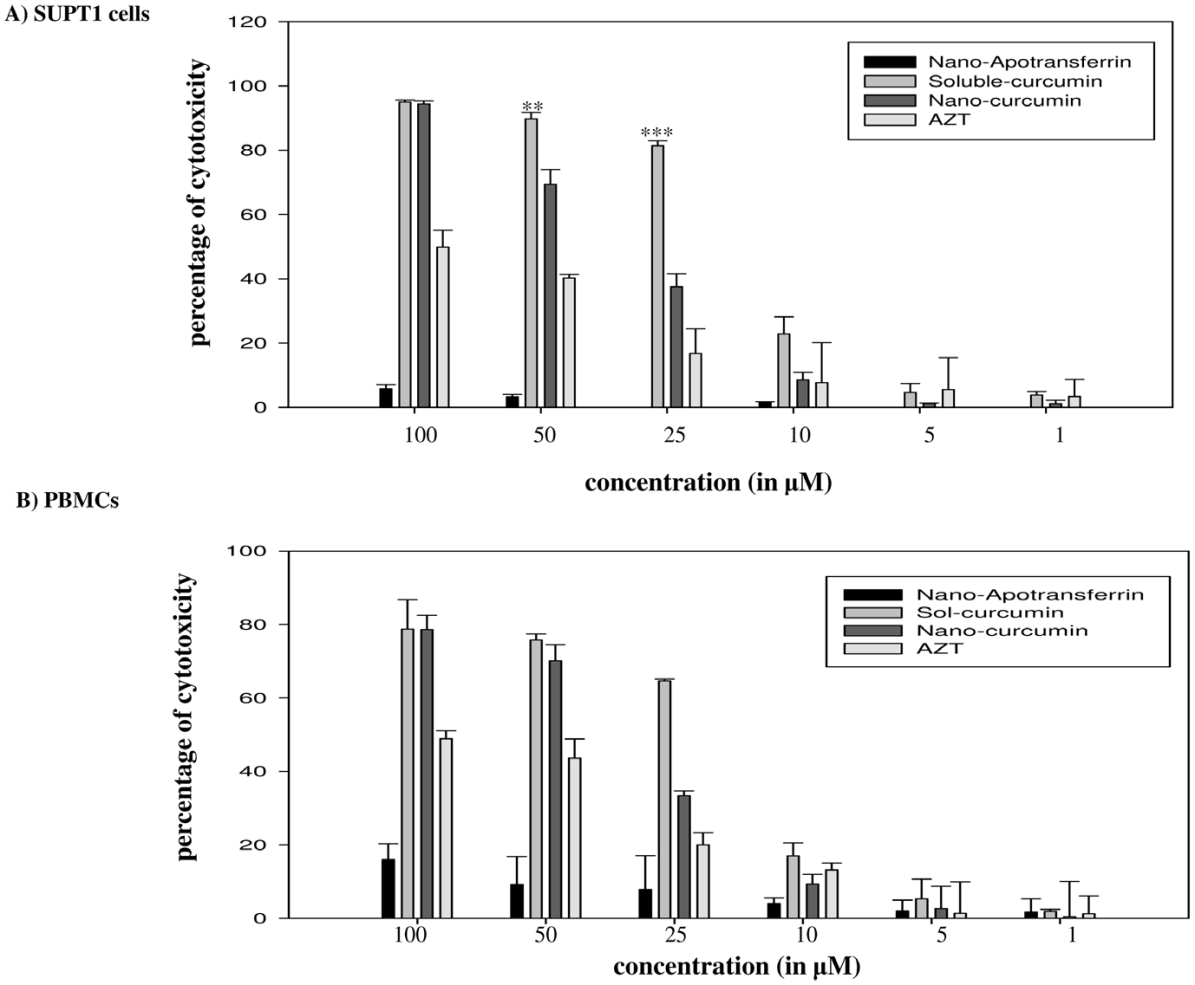
Nanoparticle formulation decreases curcumin cytotoxicity. SUPT1 cells (Panel A) or stimulated PBMCs (Panel B) were exposed to increasing concentrations (1, 5, 10, 25, 50 and 100 µM) of sol-curcumin, nano-curcumin, azidothymidine (AZT) or nano-apotransferrin (10, 50 and 100 µg) for 16 h, after which cell viability was determined by MTT assay. PBMCs were cultured in the presence of IL-2 (20 IU/ml). Cell viability in the absence of drug was defined as 0% cytotoxicity. Error bars indicate SD. ** *P*≤0.01, and ***, *P*≤0.001 compared to nano-curcumin. * indicates µg apotransferrin protein that carry equivalent molar concentration of the drug.

To determine the relative cytotoxicity of curcumin in the nano-curcumin and sol-curcumin formulations, SUPT1 cells (Fig. 5A) or stimulated PBMCs (Fig. 5B) were incubated with increasing concentrations (1, 5, 10, 25, 50 and 100 µM) of the two formulations and cell survival was estimated by MTT assay. Both sol- and nano-curcumin formulations were found non-toxic at very low concentrations (1, 5 and 10 µM). However, 25 µM concentration of sol-curcumin was extremely cytotoxic (almost 80%) while nano-curcumin at the same concentration was significantly less cytotoxic (Fig. 5). The GI_50_ of sol-curcumin is 15.6 µM, while that of nano-curcumin it is 32.5 µM in SUPT1 cells. In stimulated PBMCs, the GI_50_ is 18 µM for sol-curcumin and 38 µM for nano-cucumin. The low cytotoxicity of nano-curcumin highlights the observation that direct sol-curcumin administration is lethal to cells at concentrations above 10 µM. Notably, decreased cytotoxicity of apotransferrin encapsulated formulation occurred despite increased cellular uptake and sustained retention.

### HIV-1 neutralizing activity of nano-curcumin

**Figure 6:**
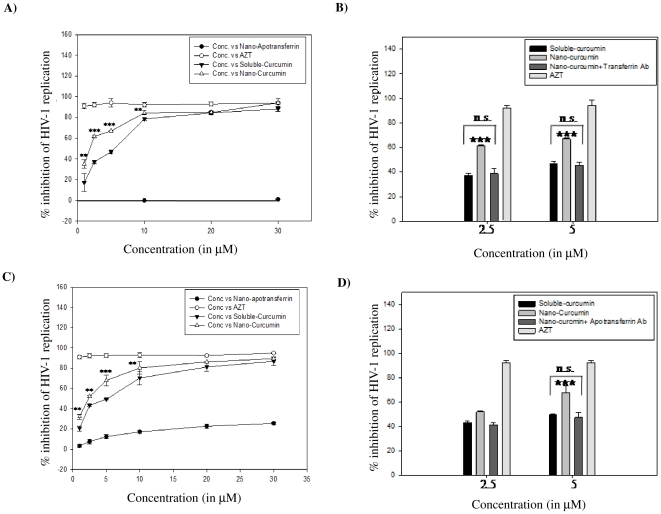
Nano-curcumin more effectively inhibits HIV-1 replication through a mechanism dependent on transferrin receptor. A & C) SUPT1 cells (Panel A) or stimulated PBMCs (Panel C) were challenged for 2 h with HIV-1_93IN101_ (1 mg p24/ml) in the presence of increasing concentrations (1, 2.5, 5, 10, 20 and 30 µM) of sol-curcumin, nano-curcumin, or nano-apotransferrin (10 and 50 µg). They were then incubated for a further 96 h, after which viral replication was measured by p24 antigen capture assay. *indicates µg apotransferrin protein that carry equivalent molar concentration of the drug. B & D) SUP-T1 cells or stimulated PBMCs were challenged for 2 h with HIV-1_93IN101_ in the presence of 2.5 or 5.0 µM concentrations of sol-curcumin, nano-curcumin or nano-curcumin in the presence of transferrin receptor antibody (100 ng/ml). After 96 h incubation, viral replication was measured by p24 antigen capture assay. In both these experiments, viral replication in the absence of drug was defined as 0% inhibition; Azidothymidine (AZT) was employed as a positive control. Error bars indicate SD. ** *P*≤0.01, and ***, *P*≤0.001 compared to sol-curcumin; n.s.: non-significant.

SUPT1 cells or stimulated PBMCs were infected with HIV-1_93IN101_ in the presence of increasing concentrations (1, 2.5, 5, 10, 20 and 30 µM) of nano-curcumin or sol-curcumin. Nano-curcumin inhibited HIV-1 replication in a dose-dependent manner (Figs. 6A and 6C). The IC_50_ of nano-curcumin is 1.75 µM, while that of sol-curcumin it is 5.1 µM in SUPT1 cells. In stimulated PBMCs, it is 5.1 µM for sol-curcumin and 2.4 µM for nano-curcumin. These data suggest that nano-cucumin is almost three-fold more potent than sol-curcumin. About 80% inhibition of HIV-1 replication was estimated at 10 µM nano-curcumin (Fig. 6A & C), a concentration at which its cytotoxicity is less than 10% (Figs. 5 A and B). Although sol-curcumin exhibits a 75% inhibition of viral replication at this concentration, its cytotoxicity is 20%. The HIV-1 neutralizing activity of nano-curcumin is significantly reduced by antibody to human transferrin receptor in both SUPT1 cells (Fig. 6B) and PBMCs (Fig. 6D), confirming that the superior ability of nano-curcumin to inhibit HIV-1 infection is dependent on cellular uptake mediated by the transferrin receptor.

### Effect of nano-curcumin on the expression of *topoisomerase IIα* and proviral DNA synthesis

**Figure 7:**
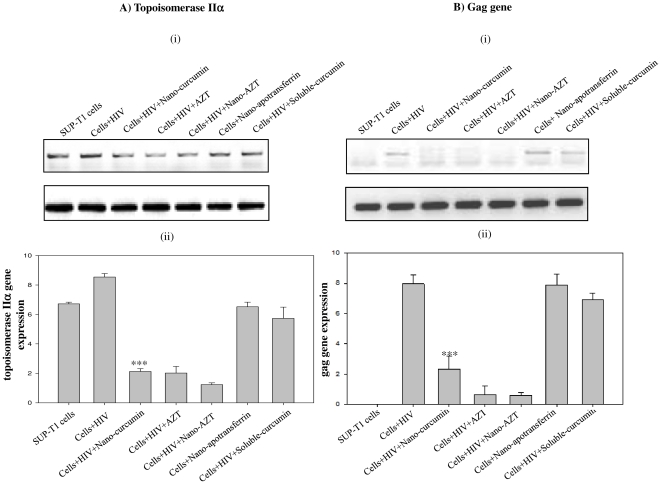
Inhibition of HIV-1 replication by nano-curcumin is due to abolished viral cDNA synthesis and/or altered topology. SUPT1 cells were challenged for 4 h with HIV-1_93IN101_ in the presence of 5 µM of sol-curcumin, nano-curcumin or nano-apotransferrin. A) The expression of topoisomerase IIα was determined by (i) semi-quantitative and (ii) quantitative-PCR. B) Quantity of viral cDNA synthesized was shown by both (i) semi-quantitative and (ii) real-time PCR using *gag*-specific primers. Template from normal SUP-T1 cells was used as negative control. Azidothymidine (AZT) was employed as a positive control and 18S was used as an internal control in both experiments. Error bars indicate SD. ***, *P*≤0.001 compared to sol-curcumin.

To determine the mechanism of nano-curcumin's anti-HIV activity in T-cells, we monitored expression of the human *topoisomerase IIα* gene. SUP-T1 cells were acutely infected with HIV-1_93IN101_ in the presence of 5 µM nano-curcumin, sol-curcumin or appropriate controls. Semi-quantitative and real-time PCR of *topoisomerase IIα* expression demonstrated that this gene was upregulated on HIV infection but was prominently down-regulated by nano-curcumin treatment (Fig. 7A). This down-regulation was not observed when the cells were treated with sol-curcumin. In a different experiment, it was found that nano-curcumin significantly inhibited proviral DNA synthesis, monitored by *gag* gene expression (Fig. 7B). Sol-curcumin had no effect on synthesis of proviral DNA.

### Nano-curcumin completely blocks HIV-1 induced inflammatory response

**Figure 8:**
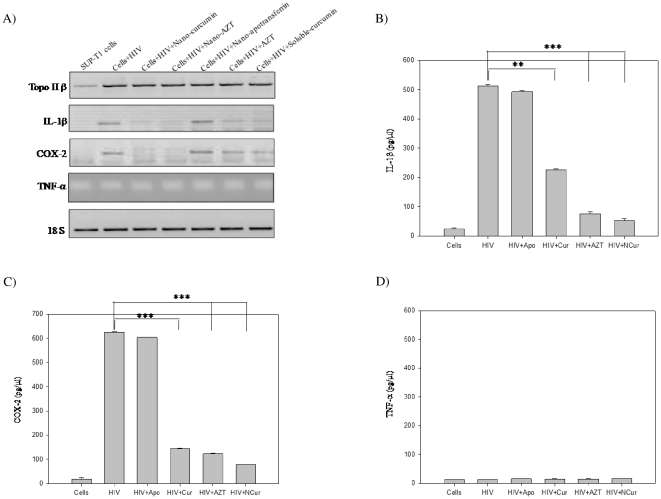
Action of nano-curcumin against virus-induced inflammatory response. A) SUPT1 cells were challenged for 4 h with HIV-1_93IN101_ in the presence of 5 µM of sol-curcumin, nano-curcumin or nano-apotranferrin. The expression of topoisomerase IIβ, IL-1β, TNF-α and COX-2 was determined by semi-quantitative-PCR. Template from normal SUP-T1 cells was used as negative control. 18S was used as an internal control in both experiments. IL-1β (Panel B), COX-2 (Panel C) and TNF-α (Panel D) were estimated using commercial kits as described in the methods section. Error bars indicate SD. ***, *P*≤0.001 compared to nano-curcumin.

HIV-1 infection enhanced expression of *topoisomerase IIβ*, *IL-1β* and *COX-2*. Treatment of infected cells with nano-curcumin significantly inhibited expression of *IL-1β* and *COX-2* (Fig. 8) as well as *topoisomerase IIα* (Fig. 7), but had no effect on the expression of *topoisomerase IIβ* or *TNF-α* (Fig. 8). The results are further confirmed by estimation of IL-1β (Fig. 8B), COX-2 (Fig. 8C) and TNF-α (Fig. 8D) levels in infected cells. These results suggest that nano-curcumin effectively blocks HIV-1-mediated inflammatory responses [21] and thus affects viral cDNA synthesis.

## Discussion

Results reported here show that curcumin-loaded apotransferrin forms spherical nanoparticles consistently in the size range 55–70 nm, which is in accord with the National Nanotechnology Initiative's definition of “nanomaterials”. Other reported formulations have typically been larger in size (usually 100–200 nm) [15], [22], although Bisht *et al*. reported a polymeric nanoparticle formulation with a size range similar to ours [23]. The spherical shape and surface characteristics of the nanoparticles, as shown by our TEM study, probably play a critical role in the cellular uptake and release characteristics displayed by this curcumin formulation *in vitro*. These particles are efficiently transported into cells through endocytosis mediated by the transferrin receptor [19], with the curcumin then being released intracellularly. Although a curcumin derivative, EF24, has been chemically linked to an endocytosis-inducing peptide [24], our nano-curcumin is to the best of our knowledge the first formulation in which cellular uptake of curcumin itself is enhanced by receptor-mediated endocytosis. The nano-curcumin formulation is especially attractive because of its simple preparation protocol that does not require either complex equipment or expensive reagents. Moreover, this delivery system is highly target-specific [19]. The simplicity of the preparation would also minimize the possibility of potentially toxic reagents being carried over into formulations for future *in vivo* studies.

Curcumin is naturally fluorescent in the visible green spectrum, thus allowing it to be located and quantified within cells. Cells treated with both sol- and nano-curcumin displayed green fluorescence, confirming successful intracellular delivery of curcumin. The greater cellular uptake and retention exhibited by nano-curcumin, as demonstrated here, addresses the problem of curcumin bioavailability. The ability of nanoparticle preparations to increase cellular uptake of curcumin has also been demonstrated by other groups [22], [23]. Since cytotoxicity is of prime concern for cellular assays, we performed our uptake and retention studies at nano-curcumin concentrations that are non-toxic to cells. Further studies showed that, despite greater intracellular concentrations, nano-curcumin exhibited much less cytotoxicity than equivalent doses of sol-curcumin.

Studies cited in the introduction demonstrate that curcumin is extremely safe and will be tolerated at dosages up to 12 g/day. This leads to the presumption that nano-curcumin will also be safe even at high concentrations, On the other hand, high-dosage AZT can cause myopathy, cardiomyopathy and hepatotoxicity associated with mitochondrial DNA depletion. As a component of HAART (highly active antiretroviral therapy) AZT causes cytopenias and lipodystrophy [25]–[29]. Similar dose-limiting adverse effects are not expected for curcumin. The present study addresses the major problem of metabolic instability and shows that nano-curcumin, even at relatively low concentrations provide a highly stable and retarded release, leading to prolonged intracellular accumulation. This prolonged exposure will reduce the needed concentration and thus the cytotoxicity, while retarded release allows longer half-life.

Studies showing that curcumin has anti-viral properties are few but reliable [30]–[32]. We now show that nano-curcumin, but not sol-curcumin, has high anti-HIV activity. Nano-curcumin drastically decreased expression levels of *topoisomerase IIα* and inhibited proviral DNA synthesis. The high levels of intracellular curcumin achieved through nano-curcumin administration may account for this previously unreported observation. Curcumin has been previously demonstrated to inhibit HIV activation and replication [30]–[33]. Mechanisms involved include inhibition of both HIV protease [32] and integrase [34]. Curcumin also inhibits Tat-mediated transactivation of the HIV long terminal repeat, which is essential for activation of latent virus [30], [31]. Another mechanism may involve inhibition of the virus's ability to upregulate the host enzyme topoisomerase II [35], which is required at the earliest stages of virus replication. Repression of topoisomerases using an inhibitor [31] or antisense nucleotides [36] results in impaired HIV-1 replication. Additionally, Topoisomerase II naturally recognizing DNA topological intermediates such as DNA curvature, flexibility, rigidity and distortion greatly influence the HIV integration event [37], [38]. Moreover, curcumin has been shown to form a complex with DNA and topoisomerase, producing DNA breaks and blocking their repair, much as etoposide does [39]. Therefore, decrease in *topoisomerase IIα*, *COX-2* and *IL-1β* levels could predictably alter the HIV-1-mediated inflammatory response, which may in turn affect the topological reorganization of cellular DNA promoted by topoisomerase IIα. These effects on DNA organization might then prevent the HIV-1 integration event. This explains the inhibition of proviral DNA synthesis, monitored through *gag* gene expression, and the consequent blockage of HIV-1 replication observed in the current study.

In summary, the ability of curcumin to block HIV replication and activation at several stages is well established. However, clinical use has been hampered by the absence of an effective dosage regime. Curcumin-loaded apotransferrin nanoparticles, through their ability to target an endocytosis-promoting cellular receptor, increase cellular uptake of the drug and enhance its ability to inhibit HIV-1 replication while simultaneously reducing cytotoxicity. Further studies using relevant *in vivo* experimental models are required. Nevertheless, this formulation appears promising as a step toward clinical usage as a multivalent antiretroviral of this well-known but underutilized therapeutic agent.

## Materials and Methods

### Preparation of stimulated PBMCs

Human blood was obtained from a healthy donor as per the approval of the Institutional Ethics Committee, University of Hyderabad. Human Peripheral blood mononuclear cells (PBMC) were isolated from blood by density gradient centrifugation using Histopaque-1077 (Sigma-Aldrich) and the cells were cultured in RPMI 1640 medium (GIBCO-BRL) supplemented with 10% FBS. PBMCs were stimulated using 10 µg/ml phytohemaggulutinin (PHA) (Sigma-Aldrich) for 2 days in the presence of IL-2 (20 IU/ml; Sigma-Aldrich) in 5% CO_2_ at 37°C. Cells were washed after 2 days and continued in culture with IL-2 for another 24 h and were stored at −70°C till further use.

### Nanoparticle preparation

Nanoparticles were prepared using a variation of the procedure described previously [19]. Ten mg of apotransferrin (Sigma-Aldrich) in 100 µl of phosphate-buffered saline (pH 7.4) was slowly mixed with 3.6 mg of curcumin (Sigma-Aldrich) in 100 µl DMSO (100 mM) and the mixture was incubated on ice for 5 min. The mixture of apotransferrin and curcumin was slowly added to 15 ml of olive oil at 4°C with continuous dispersion by gentle manual vortexing. The sample was sonicated 15 times at 4°C using a narrow stepped titanium probe of ultrasonic homogenizer (300V/T, Biologics Inc., Manassas, Virginia, USA). The sonication amplitude was 5 µm and the pulses were 30 sec long with an interval of 1 min between successive pulses. The resulting mixture was immediately frozen in liquid nitrogen for 10 min and was then transferred to ice and incubated for 4 h. The particles formed were pelleted by centrifugation at 6000 rpm for 10 min and the pellet was extensively washed with diethyl ether and dispersed in PBS. The particles' protein content was estimated by the Biuret method and the protein content was used to determine the amount of nanoparticles used for each experiment.

### Characterization of nanoparticles

Structure and morphology of the nanoparticles were investigated using a scanning electron microscope (SEM, Philips FEI-XL 30 ESEM; FEI, Hillsboro, OR, USA) operated at 20 KV, transmission electron microscope (TEM, Techni) operated at 80 KV and atomic force microscope (AFM; SPM400). For SEM the particles were gold coated, the TEM sample was prepared by fixing the sample on 200 mesh type-B carbon coated copper grid (ICON) using 2% osmium tetroxide in 50 mM phosphate buffer followed by staining with phosphotungstic acid, and the AFM sample was spin coated on a glass cover slip. Manufacturer's instructions were followed for data collection, and analysis of particles.

### Nanoparticle localization assay

SUPT1 cells obtained from the NIH-AIDS Reference and Reagents Program were used [40]. (1×10^6^) SUPT1 cells or stimulated PBMCs were seeded in 30 mm dishes (Corning Lifesciences) and treated with curcumin, either soluble or incorporated into nanoparticles at 1 and 10 µM concentrations and the cells were incubated at different time points (1, 2, 4 and 6 h). After incubation, the cells were washed thrice with phosphate-buffered saline (pH 7.4) and observed under the laser confocal microscope to analyse the amount of intracellular curcumin employing the intrinsic fluorescence of curcumin (λEx 458 nm and Em 530 nm).

### Competition of transferrin receptor antibodies with the apotransferrin-drug nanoparticles

Cells (1×10^6^) were incubated in serum-free medium for 60 min in a 12-well plate. Nano-curcumin or sol-curcumin (equivalent to 1 or 5 µM curcumin) was added to the cells in either the presence or absence of 400 ng/ml monoclonal anti-human transferrin receptor antibody (Calbiochem) and incubated for 2 h. After incubation, cells were washed thrice with phosphate-buffered saline (pH 7.4) and lysed by sonication in lysis buffer (0.1% Triton-X-100). The lysate was cleared by centrifugation at 12,000 rpm for 20 min at 4°C. Curcumin was quantified through its intrinsic fluorescent emission (λ Ex 458 nm and Em 530 nm) as measured by a fluorescence spectrometer (Shimadzu FL 2000; Shimadzu, Kyoto, Japan). In parallel, the cells were observed under a laser confocal microscope. Experiments using stimulated PBMCs were conducted in the presence of IL-2 (20 IU/ml).

### 3-(4, 5-dimethylthiazol-2-yl)-2, 5-diphenyl tetrazolium bromide (MTT) assay

SUPT1 cells or stimulated PBMCs (0.2×10^6^/well) were seeded in a 96-well plate and incubated at 37°C for 4 h in a 5% CO_2_ incubator (Forma Scientific, Marietta, OH, USA). These cells were treated with increasing concentrations of curcumin, either soluble or incorporated into nanoparticles (nano-curcumin was assessed by the amount of associated protein), and incubated for 16 h. The cells were pelleted at 1200 rpm for 10 min and resuspended in new medium. To this, 20 µl of 5 mg/ml MTT (Sigma-Aldrich) was added and incubated for 4 h. The cells were then pelleted at 1200 rpm for 20 minutes, the medium was removed, and the precipitate was dissolved in DMSO and read in an ELISA microplate reader at 570 nm.

### HIV-1 neutralization assay

SUPT1 cells or stimulated PBMCs (0.4×10^6^/ml) with 100% viability were seeded in RPMI 1640, 0.1% FBS on four 12-well plates. Increasing concentration of nano-curcumin, sol-curcumin, or nano-curcumin in the presence of anti-human transferrin receptor antibody (as indicated) were added to the cells which were then infected with HIV-1_93IN101_ at a final virus concentration equivalent to 1 ng of p24 per ml. The infected cells were incubated for 2 h at 37°C in a 5% CO_2_ incubator. The cells were then pelleted at 350× g for 10 min, the supernatant was discarded, and cells were washed with RPMI 1640 containing 10% FBS. The SUP-T1 cells were resuspended in fresh complete medium and were incubated for a further 96 h. PBMCs were incubated for 7 days. The supernatants were then collected and analyzed using a p24 antigen capture assay kit (Advanced Bioscience Laboratories, Kensington, MD, USA). The extent of infection in the absence of test compound was considered to be equivalent to 0% inhibition. Azidothymidine (AZT) was employed as a positive control. Experiments using stimulate PBMCs were conducted in the presence of IL-2 (20 IU/ml).

### qPCR analysis of *topoisomerase IIα* and *gag* expression and semi-quantitative PCR analysis of *topoisomerase IIβ*, *COX-2*, *IL-1β* and *TNF-α* expression

Genomic DNA (to monitor *gag* expression) and total RNA were isolated from treated samples using suppliers' (Qiagen, GmbH, Germany & Sigma-Aldrich respectively) protocol. cDNAs were synthesized using Superscript™ III first strand synthesis system (Invitrogen, Carlsbad, CA, USA). Primers were designed based on the gene sequences available in PUBMED nucleotide database. Multiple alignment (wherever required) was performed using the ClustalW program. For each gene, sequences of the forward and reverse primers used in each respective PCR, are as follows: *gag* Fwd: 5′ GCAGGGCCTATTGCACCAGGC 3′, *gag* Rev: 5′ GGCCAGGTCCTCCCACTCCC 3′; *topo IIα* Fwd: 5′ GGGTTCTTGAGCCCCTTCACGA 3′, *topo IIα* Rev: 5′ GTAGGTGTCTGGGCGGAGCAA 3′; 18S Fwd: 5′ GCTACCACATCCAAGGAAGGCAGC 3′, 18S Rev: 5′ CGGCTGCTGGCACCAGACTTG 3′; *topo IIβ* Fwd: 5′ GCCCAGTTGGCTGGCTCTGT 3′, *topo IIβ* Rev: 5′ GCATGGGATGAGGATCCAGGCC; *COX-2* Fwd: 5′ AACAGGAGCATCCTGAATGG 3′, *COX-2* Rev: 5′ GGTCAATGGAAGCCTGTGATG 3′; *IL-1β* Fwd: 5′ AGCTGATGGCCCTAAACAGA 3′, *IL-1β* Rev: 5′ TCTTTCAACACGCAGGACAG 3′; *TNF-α* Fwd: 5′ AGCCCATGTTGTAGCAAACC 3′, *TNF-α* Rev: 5′ CCAAAGTAGACCTGCCCAGA 3′. Real-time PCR was performed on an ABI Prism® 7500 fast thermal cycler (Applied Biosystems, Foster, CA, USA). Each sample was run in triplicate in a final volume of 25 µl containing 1 µl of template (1∶10 dilution), 10 pmol of each primer and 12 µl of Power SYBR® Green PCR master mix (Applied Biosystems). The real-time PCR results were presented as change in expression relative to control using target gene C_T_ values normalized to that of 18S gene C_T_ values based on the comparative C_T_ method [41].

### IL-1 β, PGE_2_ and TNF α assays

Cells (1×10^5^) were treated with either HIV, nano-curcumin, AZT, soluble-curcumin or apotransferrin for 4 h. Levels of IL-1β, PGE_2_ and TNF-α in the culture media were quantified using ELISA kits (R&D Systems, Minneapolis, MN, USA). The protocol was followed as per the manufacturer's instructions.

### Statistical analysis

Statistical analyses were performed using SPSS Statistics 16 (SPSS, Chicago, IL). Data are presented as mean ± S.D. Differences between groups were evaluated using Students's *t* test or one-way analysis of variance (ANOVA) with Tukey's post-hoc analysis. All experiments were repeated three times, unless otherwise specified. Values of *P*<0.05 were considered to be statistically significant.

## Supporting Information

Figure S1 **Data in** **Figure 2** **presented in a population of cells to show overall curcumin localization in a population of cells.**
(TIF)

## References

[1] Sharma OP (1976). Antioxidant activity of curcumin and related compounds.. Biochem Pharmacol.

[2] Srimal RC, Dhawan BN (1973). Pharmacology of diferuloyl methane (curcumin), a non-steroidal anti-inflammatory agent.. J Pharm Pharmacol.

[3] Mahady GB, Pendland SL, Yun G, Lu ZZ (2002). Turmeric (Curcuma longa) and curcumin inhibit the growth of Helicobacter pylori, a group 1 carcinogen.. Anticancer Res.

[4] Kuttan R, Bhanumathy P, Nirmala K, George MC (1985). Potential anticancer activity of turmeric (Curcuma longa).. Cancer Lett.

[5] Kiso Y, Suzuki Y, Watanabe N, Oshima Y, Hikino H (1983). Antihepatotoxic Principles of Curcuma longa Rhizomes1.. Planta Medica.

[6] Venkatesan N, Punithavathi D, Arumugam V (2000). Curcumin prevents adriamycin nephrotoxicity in rats.. British J Pharmacol.

[7] Srivastava R, Dikshit M, Srimal RC, Dhawan BN (1985). Anti-thrombotic effect of curcumin.. Thrombosis Res.

[8] Nirmala C, Puvanakrishnan R (1996). Protective role of curcumin against isoproterenol induced myocardial infarction in rats.. Mol Cell Biochem.

[9] Babu PS, Srinivasan K (1997). Hypolipidemic action of curcumin, the active principle of turmeric (Curcuma longa) in streptozotocin induced diabetic rats.. Mol Cell Biochem.

[10] Deodhar SD, Sethi R, Srimal RC (1980). Preliminary study on antirheumatic activity of curcumin (diferuloyl methane).. Ind J Med Res.

[11] Anand P, Kunnumakkara AB, Newman RA, Aggarwal BB (2007). Bioavailability of curcumin: problems and promises.. Mol Pharm.

[12] Cheng AL, Hsu CH, Lin JK, Hsu MM, Ho YF (2001). Phase I clinical trial of curcumin, a chemopreventive agent, in patients with high-risk or pre-malignant lesions.. Anticancer Res.

[13] Garcea G, Jones DJ, Singh R, Dennison AR, Farmer PB (2004). Detection of curcumin and its metabolites in hepatic tissue and portal blood of patients following oral administration.. Br J Cancer.

[14] Shoba G, Joy D, Joseph T, Majeed M, Rajendran R (1998). Influence of piperine on the pharmacokinetics of curcumin in animals and human volunteers.. Planta Medica.

[15] Li L, Braiteh FS, Kurzrock R (2005). Liposome-encapsulated curcumin: in vitro and in vivo effects on proliferation, apoptosis, signaling, and angiogenesis.. Cancer.

[16] Padhye S, Chavan D, Pandey S, Deshpande J, Swamy KV (2010). Perspectives on chemopreventive and therapeutic potential of curcumin analogs in medicinal chemistry.. Mini Rev Med Chem.

[17] Qian ZM, Li H, Sun H, Ho K (2002). Targeted drug delivery via the transferrin receptor-mediated endocytosis pathway.. Pharmacol Rev.

[18] Thorstensen K, Romslo I (1993). The transferrin receptor: its diagnostic value and its potential as therapeutic target.. Scand J Clin Lab Inv Supplement.

[19] Sai Krishna AD, Mandraju RK, Kishore G, Kondapi AK (2009). An Efficient Targeted Drug Delivery through Apotransferrin Loaded Nanoparticles.. PLoS One.

[20] Savarino A, Calosso L, Piragino A, Martini C, Gennero L (1999). Modulation of surface transferrin receptors in lymphoid cells de novo infected with human immunodeficiency virus type-1.. Cell Biochem and Func.

[21] Mandraju RK, Kondapi AK (2007). Regulation of Topoisomerase II a and b in uninfected and HIV-1 infected Neuroblastoma and Astrocytoma cells: Involvement of Distinct nordihydroguaretic acid sensitive inflammatory pathways.. Arch Biochem Biophys.

[22] Ratul Kumar D, Naresh K, Utpal B (2010). Encapsulation of curcumin in alginate-chitosan-pluronic composite nanoparticles for delivery to cancer cells.. Nanomed: Nanotech, Biol Med.

[23] Bisht S, Feldmann G, Soni S, Ravi R, Karikar C (2007). Polymeric nanoparticle-encapsulated curcumin (“nanocurcumin”): a novel strategy for human cancer therapy.. J Nanobiotech.

[24] Sun A, Shoji M, Lu YJ, Liotta DC, Snyder JP (2006). Synthesis of EF24-tripeptide chloromethyl ketone: a novel curcumin-related anticancer drug delivery system.. J Med Chem.

[25] Olano JP, Borucki MJ, Wen JW, Haque AK (1995). Massive hepatic steatosis and lactic acidosis in a patient with AIDS who was recieving zidovudine.. Clin Infect Dis.

[26] Sinnwell TM, Sivakumar K, Soueidan S, Jay C, Frank JA (1995). Metabolic abnormalities in skeletal muscle of patients receiving zidovudine therapy observed by ^31^P *in vivo* magnetic resonance spectroscopy.. J Clin Invest.

[27] Deveaud C, Beauvoit B, Hagry S, Galinier A, Carriere A (2005). Site specific alterations of adipose tissue mitochondria in 3′-azido-3′-deoxythymidine (AZT)-treated rats: An early stage in lipodystrophy.. Biochem Pharmacol.

[28] Mhiri C, Baudrimont M, Bonne G, Geny C, Degoul F (1991). Zidovudine myopathy: a distinctive disorder associated with mitochondrial dysfunction.. Annu Neurol.

[29] Moh R, Danel C, Sorho S, Sauvageot D, Anzian A (2005). Haematological changes in adults recieving a zidovudine-containing HAART regimen in combination with cotrimoxazole in *Cote d'lvoire*.. Antivir Ther.

[30] Barthelemy S, Vergnes L, Moynier M, Guyot D, Labidalle S (1998). Curcumin and curcumin derivatives inhibit Tat-mediated transactivation of type 1 human immunodeficiency virus long terminal repeat.. Res Virol.

[31] Li CJ, Zhang LJ, Dezube BJ, Crumpacker CS, Pardee AB (1993). Three inhibitors of type 1 human immunodeficiency virus long terminal repeat-directed gene expression and virus replication.. Proc Natl Acad Sci USA.

[32] Sui Z, Salto R, Li J, Craik C, Ortiz de Montellano PR (1993). Inhibition of the HIV-1 and HIV-2 proteases by curcumin and curcumin boron complexes.. Bioorg Med Chem.

[33] Taher MM, Lammering G, Hershey C, Valerie K (2003). Curcumin inhibits ultraviolet light induced human immunodeficiency virus gene expression.. Mol Cell Biochem.

[34] Mazumder A, Raghavan K, Weinstein J, Kohn KW, Pommier Y (1995). Inhibition of human immunodeficiency virus type-1 integrase by curcumin.. Biochem Pharmacol.

[35] Kondapi AK, Satyanarayana N, Saikrishna AD (2006). A study of the topoisomerase II activity in HIV-1 replication using the ferrocene derivatives as probes.. Arch Biochem Biophys.

[36] Bouillé P, Subra F, Mouscadet JF, Auclair C (1999). Antisense-mediated repression of DNA topoisomerase II expression leads to an impairment of HIV-1 replicative cycle.. J Mol Biol.

[37] Pruss D, Bushman FD, Wolffe AP (1994). Human immunodeficiency virus integrase directs integration to sites of severe DNA distortion within the nucleosome core.. Proceedings of the National Academy of Sciences of the United States of America.

[38] Pruss D, Reeves R, Bushman FD, Wolffe AP (1994). The influence of DNA and nucleosome structure on integration events directed by HIV integrase.. Journal of Biological Chemistry.

[39] Martin-Cordero C, Lopez-Lazaro M, Galvez M, Ayuso MJ (2003). Curcumin as a DNA topoisomerase II poison.. J Enzyme Inhib Med Chem.

[40] Smith SD, Shatsky M, Cohen PS, Warnke R, Link MP (1984). Monoclonal antibody and enzymatic profiles of human malignant T-lymphoid cells and derived cell lines.. Cancer Research.

[41] Schmittgen TD, Livak KJ (2008). Analyzing real-time PCR data by the comparative C(T) method.. Nat Protoc.

