# Supplementary figures and images for: Curcumin-Loaded Apotransferrin Nanoparticles Provide Efficient Cellular Uptake and Effectively Inhibit HIV-1 Replication In Vitro
Source: PLoS One. 2011 Aug 22;6(8):e23388. doi: 10.1371/journal.pone.0023388 (PMC3161739; doi:10.1371/journal.pone.0023388)

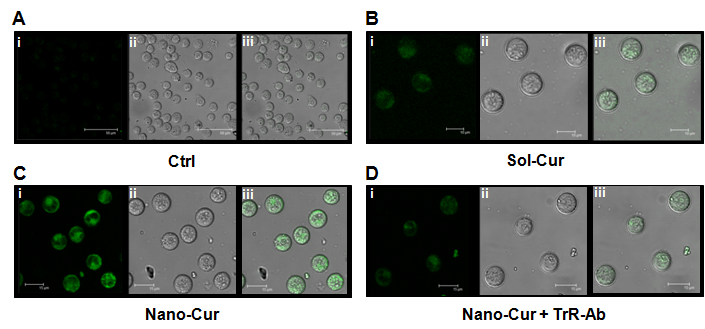

Supplement: Figure S1 — Data in Figure 2 presented in a population of cells to show overall curcumin localization in a population of cells. (TIF) [file pone.0023388.s001.tif]
